# The energy intake through regular nontherapeutic meals provision in long-term care: impact on nutritional status and related Geriatric Nutritional Risk Index

**DOI:** 10.1186/s40064-016-1763-y

**Published:** 2016-02-20

**Authors:** Baerbel Sturtzel, Ibrahim Elmadfa, Gerald Ohrenberger

**Affiliations:** Department of Nutritional Sciences, University of Vienna, Althanstraße 14, 1090 Vienna, Austria; “Haus der Barmherzigkeit”, Long Term Care Hospital, Seeböckgasse 30a, 1160 Vienna, Austria

**Keywords:** Geriatric Nutritional Risk Index, GNRI, Meal provision, Nutritional reserves

## Abstract

To investigate how the energy intake of institutionalized long-term-care patients through the regular nontherapeutic meals provision is associated with the nutritional status and the Geriatric Nutritional Risk Index (GNRI). A 9 month longitudinal, observational study. Long-term-care Hospital. 66 long-term-care patients with multiple medical conditions and solely oral food-intake. 47 (71 %) patients, predominantly women (n = 39/47), with a mean age of 83.04 (±9.58) years completed study time and 19 (29 %) deceased. At week 1 and week 36 of observation time energy intake was measured by means of three-days-weighing-records. Body composition was assessed with bioelectrical impedance analysis. Serum albumin, body weight and body height were taken from the medical report. Albumin content, body weight and height were used to calculate the Geriatric Nutritional Risk Index: GNRI = [1.489 × albumin (g/L)] + [41.7 × (weight/ideal body weight)]. Energy intake was significantly below 24 kcal/kg body weight per day. The GNRI of the deceased patients was significantly (p = 0.002) lower than the GNRI of the completers. During observation time energy-intake p < 0.001, body fat (p = 0.001) and phase angle (PA) of bio impedance measurement (p = 0.018) and likewise the GNRI (p = 0.021) of the completers decreased significantly. At the beginning and at the end of observation time energy intake correlated significantly with PA (p = 0.028/p < 0.001) and GNRI (p = 0.436/p = 0.004). Also GNRI and PA correlated significantly at the beginning (p = 0.001) and at the end (p < 0.001) of observation time. The energy intake through non therapeutic meals provision was too low for sustaining the nutritional status and likewise the GNRI. The malnourishment and the nutrition related clinical risk of the geriatric patients aggrevated during observation time.

## Background

Energy undernutrition is a prominent problem for institutionalized long-term-care patients. It is well established that a high number of frail elderly people fail to ingest an amount of food that meets their energy needs (MacIntosh et al. 2000; Cereda et al. 2013). Poor oral food consumption, with low energy intake, results in a state of under nutrition. Energy undernutrition results in a wasting of both fat and lean mass. Further, in malnourished persons, within lean mass, body cell mass is depleted relative to extracellular mass (Pencharz and Azcue 1996). Within the extracellular mass there is a contraction of plasma volume and an increase of body water. The clinical correlate of this process is the occurrence of edema (Pencharz and Azcue 1996). Therefore under nutrition have harmful clinical consequences (Muscaritoli et al. 2010). For avoiding harmful nutrition related clinical consequences it is important to assess whether the energy intake through regular non therapeutic meals provision meets the energy requirement of the geriatric patients (Anbar et al. 2014). To our best knowledge there is no sufficient information about the relationship between the oral energy intake through the regular non therapeutic meals provision and possible effects on the nutrition related clinical complications risks (Cereda et al. 2008; Volkert et al. 2013; Pedrolli and Cereda 2008).

As in geriatric clinical practice little attention has been paid to the question whether the real oral food intake has an effect on clinical outcome (Silver et al. 2008; Tamura et al. 2013; Volkert 2013) suitable criteria are needed which can be applied to assess the nutritional status and its related clinical complication risk based on biological criteria (Bouillanne et al. 2005). Mainly as is well documented that the nutrient density of the meals served is neither adapted to the poor oral intake nor to nutritional requirements of geriatric patients (Silver et al. 2008; Volkert 2013; Cereda et al. 2009). One possible explanation may be the lack of conclusive evidence as to whether improving poor nutrition by adding nutrients to common daily meals would indeed alter nutrition related clinical outcome (Cereda et al. 2008, 2009; Pedrolli and Cereda 2008).

In order to clarify how the energy intake through regular non-therapeutic meals provision influences the nutrition related clinical outcome, we measured the oral energy intake and body composition and calculated the Geriatric Nutritional Risk Index (GNRI) in respect of a causal relationship between oral energy intake, body composition and under nutrition as well as the nutrition related clinical complication risk.

## Methods

### Participants and study design

Over a period of 9 months a prospective, longitudinal, observational study was conducted in a geriatric long-term institution in Vienna/Austria. The study was approved by the ethic committee of the city of Vienna (EK 10-084-VK-NZ; 26.04.2010). Ethical guidelines were followed and an informed consent was sought from all patients. The residents of the geriatric hospital suffered from multiple chronic diseases and required assistance to perform their daily life activities. The chief physician of the geriatric hospital was asked to name patients that would be willing to participate in the planned study. As in the geriatric population many people have dementia he decided to include also patients with light dementia (MMSE > 11) (Folstein et al. 1975) when it was able to get informed consent to the study from themselves or from the procurator. Mainly these patients had an independent motivation and they were not feed by the nursing staff.

### Use of weighing records to monitor energy intake

The energy-intake of the geriatric patients was assessed by 3-day-weighing records at week 1 and week 36 of observation time. The weighing records were conducted by trained staff, who weighed the plates with the meals before and after the participants` food intakes during the course of the day. The calculation of the energy intake was accomplished with nut.s^®^ nutritional software based on the German Food database BLS 3.01. For the calculation of the energy intake the mean (±SD) of the 3-day-weighing records was used.

### Anthropometric measurements

Body height (m) and body weight (kg) measurements were conducted by clinical staff. When possible, body height was measured free-standing with a flexible measuring tape (cm) for assessing distorted vertebral column, if not, height was measured reclined, also with a flexible measuring tape (cm). Body weight was measured in subjects wearing light clothes and bare-footed with a calibrated scale.

BMI was calculated according to the formula BMI = body weight in kg/body height in m^2^.

### Bioelectrical impedance measurements

Bioelectrical impedance analysis (BlA) was used to assess changes in body composition. Therefore we measured changes in body lean and fat mass plus phase angel (PA) with bioelectrical impedance analysis. Phase angel (PA), body lean mass and fat mass were measured with Bodystat 1500^®^MDD in a multi-frequency (5/50 kHz) technique. Measurements were taken using a standard hand-to-foot tetra-polar technique with participants in supine position by a trained technician in accordance with the manufacturer’s guidelines. Raw impedance measurements of resistance, R and capacitance and phase angel (PA) were recorded. The PA component was calculated using the equation: PA (degrees) = arctan (Xc/R) × (180/Pi).

### Laboratory assessments

Serum albumin was assessed at the geriatric hospital’s central laboratory; the data were taken from the medical report at baseline (week 1) and at the end (week 36) of observation-time.

### Calculation of the Geriatric Nutritional Risk Index (GNRI)

The GNRI was calculated using measurement of serum albumin, body weight and body height measured as follows (Bouillanne et al. 2005):

$${\text{GNRI}} = \left[ {1.489 \times {\text{albumin}}({\text{g}}/{\text{L}})} \right] + \left[ {41.7 \times ({\text{weight}}/{\text{ideal}}\;{\text{body}}\;{\text{weight}})} \right].$$

The ideal weight was defined using the Lorentz equations (Bouillanne et al. 2005), as follows:

$${\text{Height}}\,({\text{cm}}) - 100 - \left( {[{\text{height}}\;({\text{cm}}) - 150]/4} \right)\;{\text{for}}\;{\text{men}},\;{\text{or}}$$

$${\text{Height}}\,({\text{cm}}) - 100 - \left( {[{\text{height}}\;({\text{cm}}) - 150]/2,5} \right)\;{\text{for}}\;{\text{women}}.$$

When the patient’s weight exceeded the ideal weight, parameter “(weight/ideal weight)” was set to 1 (Bouillanne et al. 2005).

The GNRI was stratified according to the categorization proposed by Cereda et al. (2009, 2013). The results of our analysis were categorized using the following cut-off values: high nutritional risk = GNRI < 92; low nutritional risk = GNRI 92–98; no nutritional risk = GNRI > 98.

### Data analysis

Data were analyzed using SPSS (Windows version 18.0, IBM-SPSS, Inc., Chicago, IL). Kolmogorov–Smirnov-Test was used for establishing normal distribution. As the sample size was small and body weight (p = 0.020) and body mass index (BMI) (p = 0.038) were not normally distributed we chose non-parametric tests for the statistical analysis. We show means and standard deviations or counts and percentages. For continuous variables the non-parametric Wilcoxon signed-rank test for repeated measurements was used to establish the differences within the groups. The non-parametric Mann–Whitney-U-Test for independent samples was used to establish the differences between the groups. Significantly differences between categorical variables were established by using Chi squared test. The bivariate Pearson test was used to establish correlations. A p value of <0.05 was considered statistically significant for all analyses.

## Results

The chief physician selected 232 geriatric patients. Eighty-five patients (74 female and 11 male) gave their consent to take part in the study. Nineteen patients with a mean age of 87.8 (±4.77) years with taking nutritional supplements or receiving enteral or parenteral feeding were excluded. Of the 66 patients enrolled in the study 19 geriatric patients with a mean age of 88.7 (±6.8) years died during observation time. Forty-seven patients [41 female (87 %) and 6 men (13 %)] with a mean age of 83.4 (±10.8) years and solely oral food intake completed the study.

At the beginning of the study the Geriatric Nutritional Risk Index (GNRI) of the deceased was significantly below the GNRI of the completers (see Table 1). Total energy intake through the non-therapeutic meals provision was low and below 24 kcal/kg body weight per day (D-A-CH 2000; Elmadfa and Leitzmann 2015). Furthermore the energy intake decreased significantly (p = 0.001) between week 1 and week 36. Likewise the phase-angle of bio impedance analysis (p = 0.018) and the GNRI (p = 0.021) of the completers decreased significantly (see Table 2). The percentage of the completers with a high nutrition related clinical risk increased about 6.6 % and with a low risk about 2.2 % (see Table 3). The energy intake was too low for sustaining the nutritional reserves of the body, which was also indicated by the decreasing fat mass (p = 0.001) (see Table 2). That the small energy intake through the non-therapeutic meals provisions have its impact on the worsening of the nutritional status was indicated at the beginning and at the end of observation time by the significant correlations between the energy intake and the phase angle (p = 0.028/p < 0.001), between the energy intake and the GNRI (p = 0.436/p = 0.004) and between the GNRI and the phase angle (p = 0.001/p < 0.001) of the completers (see Table 4).

**Table 1:** Comparison of patients’ characteristics (mean ± SD) of deceased with completers at week 1 of observation time

	Deceased (N = 19)	Completers (N = 47)	p value^1^
Body weight (kg)	61.5 (±10.4)	67.1 (±16.0)	0.376
Body height (cm)	160.6 (±8.0)	160.7 (7.3)	0.806
BMI (kg/m^2^)	23.6 (±4.1)	25.8 (±5.4)	0.139
Albumin (g/dl/serum)	3.13 (±0.55)	3.44 (±0.41)	0.003*
GNRI	84.7 (±6.4)	91.9 (±6.8)	0.002*
Phase-angle	2.93 (±0.83)	3.32 (±0.73)	0.169
Lean Body Mass (%)	57.5 (±7.0)	54.8 (±7.8)	0.297
Total Body Water (%)	52.0 (±7.4)	48.2 (±7.2)	0.143
Body Fat Mass (%)	42.4 (±7.0)	45.2 (±7.8)	0.260

^1^Significantly differences between mean (±SD) of deceased and completers at week 1 were tested by using Mann–Whitney-U-Test

**Table 2:** Evaluation of mean (±SD) energy-and protein intake, bodyweight and BMI, Albumin and GNRI and bioelectrical impedance analysis of geriatric patients at week 1 and week 36

	Week 1 (N = 47) (mean ± SD)	Week 36 (n = 47) (mean ± SD)	p value^1^
Energy intake (kcal/d)	1204 (±238)	1002 (±283)	0.000*
(kcal/kg/BW/d)	18.6 (±5.7)	15.9 (±5.4)	0.001*
Protein intake (g/d)	46.1 (±13.9)	36.0 (±12.0)	0.000*
(g/kg/BW)	0.65 (±0.22)	0.55 (±0.19)	0.000*
Body weight (kg)	67.1 (±16.0)	65.1 (±16.8)	0.047*
Body height (cm)	160.7 (±7.3)		
BMI (kg/m^2^)	25.8 (±5.4)	25.1 (±5.3)	0.048*
Albumin (g/dl/serum)	3.44 (±0.41)	3.32 (±0.51)	0.052
GNRI	91.9 (±6.8)	89.8 (8.3)	0.021*
Phase-angle (°)	3.32 (±0.73)	3.08 (±0.71)	0.018*
Lean body mass (%)	54.84 (±7.8)	57.4 (±7.85)	0.002*
Total body water (%)	48.2 (±7.2)	51.5 (±6.5)	0.000*
Body fat mass (%)	45.2 (±7.8)	42.3 (±7.9)	0.001*

^1^Significantly differences between means of completers of week 1 and week 36 were tested by using Wilcoxon-Rang-Test

* Week 1 and week 36 = significantly different when p value <0.05

**Table 3:** Percentage (%) of non-completers (n = 19) and completers (n = 47) at high, low and no nutrition related clinical risk according the GNRI categorization at week 1 and of the completers at week 36

	Non-completers %	Completers %		p value^1^
	Week 1	Week 1	Week 36	
High risk (GNRI < 92)	91.7	47.8	53.3	
Low risk (GNRI 92–98)	8.3	28.3	31.1	
No risk (GNRI > 98)	–	23.9	15.6	p < 0.001*

^1^Significantly differences between the categorical variables of GNRI-categorization were Chi squared tested

* Significantly differences exist when p < 0.05

**Table 4:** Correlations of the Geriatric Nutritional Risk Index (GNRI) with the protein-and energy intake, likewise the correlations of phase angle with protein-and energy intake and the correlations of GNRI with phase angle

GNRI/protein intake (g/day)^a^	Correlation (r)^e^	p value	Phase angle/protein (g/d)	Correlation (r)	p value
Week 1	0.156	0.305	Week 1	0.184	0.232
Week 36	0.516	0.000*	Week 36	0.573	0.000*

GNRI/energy intake (kcal/d)^b^			Phase angle/energy (kcal/d)		
Week 1	0.120	0.436	Week 1	0.331	0.028*
Week 36	0.421	0.004*	Week 36	0.589	0.000*

GNRI/protein intake (g/kg/BW/d)^c^			Phase angle/protein (g/kgBW/d)		
Week 1	0.029	0.853	Week 1	0.242	0.113
Week 36	0.358	0.017*	Week 36	0.455	0.003*

GNRI/energy intake (kcal/kg/BW/d)^d^			Phase angle/energy (kcal/kgBW/d)		
Week 1	−0.059	0.702	Week 1	0.248	0.105
Week 36	0.201	0.190	Week 36	0.386	0.013*
GNRI/phase angle					
Week 1	0.469	0.001*			
Week 36	0.609	0.000*			

* Significantly correlations exist when p < 0.05

^a^Protein intake = protein (g) per day)

^b^Energy intake = energy (kcal) per day

^c^Protein intake = protein (g) per kg body weight per day

^d^Energy intake = energy (kcal) per kg body weight per day

^e^Correlations were tested by using the Pearson test

## Discussion

In this prospective longitudinal observation study we measured and calculated different markers to determine whether the oral energy intake through regular non therapeutic meals provision of geriatric patients has an impact on the nutritional status and especially on the nutrition related clinical complication risk.

The examined patients were multi morbid and severely ill which was reflected by the fact that the average phase angle of the bio impedance analysis was substantially below the existing age-specific reference values (Wirth et al. 2009).

The results of the weighing protocols highlighted that the energy intake through the free meal choice of the non-therapeutic meals provision was too low to meet the energy requirements of the geriatric patients. At the beginning as well as at the end of observation time the measured energy intake was significantly below the recommended intake for sustaining the resting energy expenditure (Elmadfa and Leitzmann 2015) and for preventing energy undernutrition. Moreover, during observation time the nutrient intake even declined further and energy undernutrition progressed. This fact was reflected by the poor nutritional status and the altering body composition of the geriatric patients. The energy reserves of the body, the body fat mass, declined significantly. At the same time the percentage of lean body mass did not changed while total body water increased significantly. We assumed that the total body water retention masked a progressive reduction of body cell mass. Like Pencharz and Azcue (1996) we conclude that the occurrence of edema, which is an effect of energy under nutrition, masked the wasting of lean body lean mass.

Pencharz and Azcue (1996) documented these effects of energy under nutrition in malnourished children. But the actually literature told us, that in old multi morbid patients these effects of under nutrition alone for assessing a worsening of the nutritional status of geriatric patients can be challenged. Therefore the concept that the phase angle of bio impedance analysis is a good marker for assessing malnutrition in this multi morbid cohort is now approved (Wirth et al. 2009; Slee et al. 2015; Wirth and Miklis 2005; Norman et al. 2012).

During the course of our study the phase angle dropped as did the body fat mass. Both occurred in parallel to a decreasing energy intake. It was obvious that the declining phase angle reflected the insufficient energy intake and the worsening of the nutritional status which was poor from the beginning on. The energy intake through oral food intake was inadequate to maintain the nutritional reserves of the body. Therefore, as also reported by other authors (Donini et al. 2008; Dupertuis et al. 2003), we observed that a great number of the geriatric patients did not eat enough to cover their minimum energy needs.

Above all, we conclude that this state of small energy intake and resulting under nutrition obviously has its impact on the nutrition related clinical outcome. This is further supported by the findings that the GNRI-Index of the non completers was significantly lower than the GNRI-Index of the completers. The lower GNRI of the deceased geriatric patients, measured at the beginning of the study, indicated that the clinical outcome of them was more influenced by the nutritional status than the clinical outcome of the completers. In this line we consider that the falling energy intake and the mounting poor nutritional status cause a worsening of the related clinical outcome and finally contributed to the mortality risk. These considerations could be supported by interpreting the correlation between the GNRI-Index and the phase angle of bio impedance analysis, the correlation between the GNRI-Index and the energy intake and the circumstance that only 15.6 % of the completers had no nutrition related clinical risk at the end of observation time. Therefore we conclude that if the energy content of the regular nontherapeutic menus is not adapted and balanced to the energy uptake and requirements of the patients the nutrition related clinical risk will increase over the stay in the long-term-care hospital.

Hence, in order to stabilize the health status of the geriatric patients we have to find ways to adapt the nutrient density of the served meals to the poor oral intake and to the nutritional requirements of the patients to avoid the harmful consequences of a poor nutritional status. The same conclusions were drawn by other investigators in the few comparable published studies. They concluded that improving the nutritional quality of long-term-care patients regular non therapeutic menus is worthwhile (Cereda et al. 2013; Donini et al. 2008; Iff et al. 2008), especially as intervention studies suggested that efforts to improve the oral intake of geriatric patients by increasing the nutrient density of the daily meals, can rectify the imbalance between oral nutrient intake and demands (Anbar et al. 2014; Volkert et al. 2013; Tieland 2012). In our work we were able to show, that a long-term, selected limited nutrient improvement of the daily meals provision with easily consumed, traditional and calorie-rich food served through the catering-service-system of the geriatric hospital was able to improve the energy intake and in consequence to improve the nutritional status of the geriatric patients (Sturtzel et al. 2013).

Nevertheless, our study here has some limitations. It is nearly impossible to clearly distinguish between non nutritional and nutritional causes for decreased serum albumin and body weight loss. This could be relevant as in multi morbid patients it is well documented that disease and nutrition interact and disease may cause secondary malnutrition or malnutrition may be unfavorably influenced by underlying diseases (Abellan van Kan et al. 2009; Kagansky et al. 2005) and vice versa. Additionally our sample size was small. But it is difficult to find geriatric patients which are willing or in the position for giving informed consent. For example, many are not interested to take part in the study or they are deep demented.

However, the added value of this longitudinal observational study on the energy intake of geriatric patients was the use of the well validated GNRI-Index to also assess the clinical relevance of the energy intake through non-therapeutic meals provision. We clearly demonstrated that the energy intake of multi morbid geriatric patients through regular non therapeutic meals provision was too low for sustaining the nutritional reserves and to avoid an aggravation of the nutrition related clinical risk.

## Conclusion

Our results reinforce the relevance of nutrition in the outcome of geriatric clinical practice. This longitudinal observational study could show that the nutritional status decreased and the nutrition related clinical complication risk increased when the energy intake through the non-therapeutic meals provision was too low. In geriatric clinical practice more attention should be paid to the composition and the nutrient density of the daily served meals when a poor nutritional status or a low Geriatric Nutritional Risk Index became obvious in geriatric patients.
